# Giggle incontinence: A rare condition with a successful management

**DOI:** 10.1016/j.eucr.2022.102033

**Published:** 2022-02-16

**Authors:** Ihab Ibrahim Abdelmonem, Mohammed Khalid Khan, Omar Abdulrahman AL muhammadi, Aahid Rozan, Mahmoud Jamil Merdad, Maged Khalid Baesia

**Affiliations:** a Department of Urology, King Abdullah Medical Complex, Jeddah, Saudi Arabia; b Batterjee Medical College, Jeddah, Saudi Arabia

**Keywords:** Giggle incontinence, Methylphenidate, Case report, Laughter

## Abstract

Giggle incontinence (GI) is a rare condition characterized by involuntary loss of urine during giggling or laughing. Due to associated social embarrassment, it is difficult to recognize and, without a clear management plan, to treat. In this case report, we present a 16-year-old girl with successfully treated GI, discussed with a review of the related literature.

## 1 Introduction

Giggle incontinence (GI) is an involuntary episode of urine loss induced by an episode of laughter. The scarce reported literature indicates a higher prevalence in adolescent girls. The pathogenesis of GI is poorly understood. We present here the case report of a 16-year-old female who suffered from urine incontinence whenever she laughed. Subsequently, she was treated with methylphenidate, which has shown excellent safety and control of the patient's condition on follow-up. Afterwards, we report the case after obtaining informed consent from the patient.

## 2 Case presentation

A 16-year-old girl was referred to our urology clinic, from a primary care center, complaining of total unstoppable urinary incontinence when laughing since childhood. She is medically free, has regular menstruation since age 11, and does not complain of any other urinary symptoms. She had previously been prescribed an anticholinergic agent elsewhere but without clinical response.

Her physical examination, standard urine and blood workup, and ultrasound examination were all unremarkable. We counseled her for urodynamic assessment and the study showed normal parameters for bladder filling with non-demonstrated detrusor overactivity or stress incontinence. Her treatment plan was based on the rare available literature which demonstrated anecdotal but successful treatment with methylphenidate. We started with an initial dose of 12 mg oral methylphenidate every morning for one month. On the first follow-up visit, she was happy with the almost complete disappearance of symptoms, and she tolerated the drug well. We advocated for her to keep on methylphenidate for an additional three months. Follow-up visits over the next year demonstrated the efficacy and safety of the medication. The patient had taken the medication for a total of nine months, after which we managed a tapering-down plan for one month. On her next follow-up visit, she was completely continent after discontinuation of the medicine for two months.

## 3 Discussion

Giggle incontinence, is a relatively rare condition characterized by involuntary and typically complete bladder emptying specifically in response to giggling or laughing, with otherwise normal bladder function when the child is not laughing.^1^ It appears to mostly affect early or mid-pubescent girls, and may be associated with a strong female family history of this syndrome. This condition can drastically affect quality of life due its irritating nature and due to fear of social embarrassment. GI is a distinct condition from stress incontinence, in which a small amount of urine leaks with sneezing, coughing, straining or other movements that increase intra-abdominal pressure, thereby causing increased pressure on the bladder; careful differentiation and exclusion of this form of incontinence should be made before the diagnosis of GI can be established.^1^

The pathophysiology of GI is unclear. However, among the various studies conducted investigating the etiology of giggle incontinence, two differing explanations, neurogenic and non-neurogenic, have been proposed.^2^

The predominant theory is that giggle incontinence is primarily or at least partially centrally-mediated. It is postulated to be related to a receptor imbalance of the cholinergic and monoaminergic systems, showing resemblance to cataplexy in that laughter induces loss of muscle tone, which therefore emphasizes treatment with methylphenidate.^2^ Methylphenidate (MPH) is a central nervous system stimulant commonly used to treat attention deficit hyperactivity disorder (ADHD). Two studies evaluating the effectiveness of methylphenidate in GI have found methylphenidate to have a high success rate, resulting in a full response of complete cessation of wetting in 80% and 100% of trial participants, among sample sizes of 15 and 9 patients respectively.^3^^,^^4^ Treatment duration ranged from 2 months to more than 3 years. However, relapses after treatment cessation were reported in some patients and the time to relapse after stoppage of the medication was not evaluated. The occasional long duration of treatment needed for complete symptom resolution (up to 3 years or more) and the tendency to relapse after treatment cessation, especially if the treatment was only given for a shorter time (i.e., two months), makes it unclear as to whether patients treated with methylphenidate were actually cured of, or simply outgrew, the condition.

Other studies have postulated that urologic dysfunction is the cause of GI. As such, they recommend a more traditional approach of timed voiding/bladder retraining, improving sphincter contraction through pelvic floor muscle exercises, and improving muscle recruitment using biofeedback techniques; this treatment strategy has found modest success.^2^

A recent retrospective study of children who failed to respond to behavioral urotherapy compared the efficacies of biofeedback therapy and methylphenidate in GI over a one-year period and found similar success rates for both in the first, third, and sixth month of follow-up. However, the 12th month's assessment found fewer patients who achieved a full or complete response with MPH (55.6%) compared to biofeedback (94.1%). Irritability, agitation and sleep disturbances were side effects that necessitated discontinuation of MPH in three patients.^5^ This study was limited, however, by the duration of MPH treatment, which lasted for only the first three months of the 12-month period; a longer duration of treatment with MPH may have yielded better results.

Based on the studies mentioned above, it is evident that different studies support different treatment modalities. Due to some limitations in these studies, including small sample sizes, the most suitable treatment of GI is yet to be conclusively decided. Additionally, the natural history of untreated GI and whether or not medications shorten its course is also not known or well documented. Thus, there is a need to initiate a large multi-center longitudinal study or clinical trial with longer follow-up periods to help draw a conclusion on the most appropriate management of this mysterious condition.

## 4 Conclusion

Giggle incontinence is an unusual condition in which laughter stimulates involuntary micturition. Due to its uniqueness, management strategies, beyond standard behavioral urotherapy, are varied but not well-established and essentially include medications such as methylphenidate, which targets the presumed mainly-neurogenic pathophysiology of GI, and biofeedback techniques, which target a more urologic origin. While the condition is relatively harmless, it may cause significant annoyance, discomfort, and can greatly impact his/her quality of life, thus highlighting the importance of treatment.

## Funding

This research did not receive any specific grant from funding agencies in the public, commercial, or not-for-profit sectors.

## Consent for publication

Written informed consent was obtained from the parents for publication.

## Declaration of competing interest

None.
